# Impacts of casinos on key pathways to health: qualitative findings from American Indian gaming communities in California

**DOI:** 10.1186/s12889-016-3279-3

**Published:** 2016-07-22

**Authors:** Stephen R. Kodish, Joel Gittelsohn, Vanessa M. Oddo, Jessica C. Jones-Smith

**Affiliations:** Department of Nutrition, Harvard T.H. Chan School of Public Health, 677 Huntington Ave, Boston, MA 02115 USA; Department of International Health, Center for Human Nutrition, Johns Hopkins Bloomberg School of Public Health, 615 N. Wolfe St, Baltimore, MD 21205 USA

**Keywords:** Social policy, Health impacts, Built environment, Qualitative research, American Indian health, Casinos, Nutrition, Physical activity

## Abstract

**Background:**

Three decades ago, casino gaming on sovereign American Indian lands was legalized with differential economic and social implications. While casinos have improved the incomes of tribal communities, there have been both positive and negative findings in relation to health impacts. We sought to understand the perceived pathways by which casinos impact individual and community health through voices of the community.

**Methods:**

We conducted semi-structured, interviews with tribal leaders (*n* =12) and tribal members (*n* =24) from tribal communities (*n* = 23) representing different regions of California. We inductively analyzed textual data drawing from Grounded Theory, first using line-by-line coding to identify analytic categories from emergent themes in consideration of the study objective. Then, focused codes were applied to identify salient themes, which we represented through exemplar quotes and an overall conceptual framework. Data were managed and coded using Dedoose software.

**Results:**

American Indian-owned casinos are perceived to influence the health of tribal communities through three pathways: 1) improving the tribal economy 2) altering the built environment, and 3) disrupting the the social landscape. Forming these pathways are a series of interrelated health determinants. Improvement of the tribal economy, through both job creation for tribal members and improved tribal cash flow, was perceived by participants to both influence health. Specifically, improved cash flow has resulted in new wellness programs, community centers, places for recreation, and improved social services. Higher disposable incomes have led to better financial stability, increased access to healthy food, and more opportunities for physical activity. Yet, higher disposable incomes were perceived to also contribute to negative health behaviors, most notably increased drug and alcohol abuse. Casinos were also perceived to alter built environments, resulting in increased availability and access to unhealthy food. And to a lesser extent, they were perceived to disrupt the social landscape of communities with impacts on tribal community value systems.

**Conclusions:**

Casino environments improve economic conditions of tribal communities, but present important social and public health challenges. Policy makers at federal, state, and tribal levels should consider the perceptions of tribal members and leaders when determining policies in light of casino development.

## Background

Populations with lower incomes experience higher risk for many diseases compared to their higher-income counterparts [1]. To investigate the extent to which income or other economic resources may causally impact health, a small number of experimental and quasi-experimental studies have been developed [2–10]. Specifically, a number of these studies have considered American Indian-owned casinos as a natural experiment to study the impact of an income shock on health [2, 3, 6, 9–11]. American Indian populations are particularly important population for which to estimate the impacts of an income shock since they are disproportionately burdened by poverty and they share a disproportionate burden of mental health disorders [12–14], tobacco usage rates [15], food insecurity levels [16], chronic diseases [14, 17–19], and drug and alcohol abuse [12, 14].

American Indian-owned casinos were legalized by the United States federal government for the specific goal of promoting poverty alleviation and self-sufficiency for American Indians. The intended purpose was for tribal communities to use casino revenues to fund their own governments, invest in needed community infrastructure, provide jobs for their members, and make any other changes deemed necessary to improve overall community welfare.

Several studies have shown that casinos have indeed succeeded at improving the incomes of American Indians [6, 9, 20, 21]. Consequently, these findings have led health researchers to use this natural experiment to study whether the increase in economic resources is associated with improved health. To our knowledge, there have been 7 studies to examine this relationship, with mixed health impacts reported. Nationwide studies taking a natural experiment approach have found casinos or income from casinos to be associated with a greater decrease in total mortality, obesity, diabetes, smoking and binge drinking among American Indian adults [6, 9]. Using data from approximately 100 tribes in California, Jones-Smith and colleagues [21] found that living nearby a casino was associated with decreased risk of childhood overweight and obesity. Four additional studies have used data from the Eastern Band of Cherokee in North Carolina and found that among children for whom the opening of a casino took them from being poor to non-poor, behavioral problems decreased to the levels seen in those children who were never poor [2]. Costello followed these children into adulthood and found persistent decreases in risk of psychopathologies and decreases in risk for alcohol or cannabis abuse [2]. On the contrary, Akee and colleagues found that the children who were poorest at baseline experienced a significant increase in risk for obesity as young adults [10]. Similarly, Bruckner and colleagues found that in the months when the casino typically issued dividend payments to tribal members (June and December), accidental deaths among adults increased [11].

With the exception of qualitative work examining methamphetamine users among the Eastern Band of Cherokee community, to our knowledge, no other studies have used in-depth qualitative data to explore the pathways by which American Indian-owned casinos may result in these health impacts. This fact is particularly important since these studies rely on natural experiments and typically employ pre-existing data collected for other purposes, while providing very little opportunity for any quantitative testing of proposed mediators [22].

Understanding how tribal member perceptions match or do not match the quantitative findings of casino health impacts is important for tribal councils and public health practitioners who are tasked with making resource allocation decisions related to gaming revenues. To more fully understand the pathways by which casinos might impact health we sought to understand how tribal leaders and members perceive the influence of casinos on determinants of their individual and community health.

## Methods

### Study context

California is home to nearly 300,000 American Indians, more than any other state [23]. There are 109 federally recognized tribes and 104 different tribal lands [24]. These tribal lands tend to be very small areas of land and they are interspersed throughout the state. The number of American Indians living on these tribal lands ranges from 0 to approximately 2,500 [23]. Fifty-eight tribes in the State own casinos, which range in size from <100 to >2,000 slot machines, a commonly-used metric for casino size.

The terms for gaming on tribal lands vary by state. In California, tribes pay into the state’s General Fund based on the number of slot machines in their casinos [25]. Uniquely, tribes with large casinos also pay into a separate fund called the Revenue Sharing Trust Fund (RSTF). The RSTF funds are then redistributed to tribes without casinos or with small casinos (<350 slots) [25, 26]. This mechanism was primarily designed to alleviate between-tribe income inequality because a small subset of tribes operate highly profitable casinos. Currently, each tribe that either does not own a casino or owns a small casino (<350 slots) in California receives $1.1 million annually from the RSTF and has the autonomy to decide how to spend this money [27]. Sometimes tribes will distribute funds among members as “per capita payments” whereas others will invest in community development projects. Per capita, payments, from either a casino or the RSTF (henceforth, “per capita” is used in reference to either type of payment), thus vary in amount by tribal membership and on casino profit size (for gaming tribes) [25, 26].

### Research design

Building off findings from a natural experiment that examined the impact of casinos on the weight-related health of American Indian women and children in California [21], we used an emergent, qualitative study design to understand how tribal leaders and members perceived casino impacts on individual and community health [28]. Characterized by an emergent and iterative design [29] this qualitative study utilized semi-structured interviews as the primary data collection method. This method was chosen for its open-ended question format, ability to collect rich information, and potential to generate thick descriptions for best answering the research questions [30].

### Sampling and data collection

The interviews were conducted between May 2014 and April 2015 among tribal leaders (*n* = 12) and tribal members (*n* = 24) from 23 of the 109 (21 %) different federally-recognized tribes in California [24]. These two types of participants were interviewed in order to triangulate data sources as a strategy to increase the credibility of our findings [31]. We used criterion-based, purposive sampling to identify information-rich participants who could provide deep insights about both themselves and their own communities during the one-on-one interviews [32, 33].

Tribal leaders and members were eligible to participate if they met the following criteria: 1) spoke English fluently, 2) were 18 years or older, and 3) had tribal affiliation or had a child who had tribal affiliation, and 4) lived on tribal lands or in a neighboring community. We also purposively stratified our sample [34] to find a balance of participants from each of the three geographic regions of California, from varying tribal sizes, and with gaming (i.e., tribes with casinos) or non-gaming affiliation (i.e., tribes without casinos). Some eligible participants were not available for interviews due to scheduling conflicts and one participant dropped out of the study after enrollment. Table 1 outlines participant and tribal demographic information.

**Table 1:** Demographic Characteristics of Respondents

	Tribal Leaders	Tribal Members	Tribes
	(*n* = 12)	(*n* = 24)	(*n* =23)
Sex			
Male	4	4	---
Female	8	20	---
Gaming Affiliation			
Gaming	9	15	14
Non-Gaming	3	9	9
Geographic Location			
Northern California	4	9	7
Central California	7	8	8
Southern California	1	7	8
Number of Slot Machines in Casino^a,b^			
< 350 Slot Machines	7	8	7
350–1,000 Slot Machines	1	3	2
> 1,000 Slot Machines	1	4	5
Number of Tribal Members			
< 200 members	2	1	3
200–1,000 members	9	12	14
> 1,000 members	1	11	6

^a^Excludes participants of non-gaming tribes (*n* = 9)

^b^We used the following casino size designations based on number of casino slot machines: (<350 slot machines = small casino); (350–1,000 slot machines = medium casino); (>1,000 slot machines = large casino)

After gaining informed consent, data were collected by the principal investigator (JJS) and graduate student research assistants who were trained in qualitative research theory and interviewing methodology. Prior to interviews, the research team had not met all of the participating tribes in person, but efforts were made to build rapport through ‘ice breaker’ questions which were part of the interview guides used to elicit rich responses through open-ended questioning and probing [32]. Interviews lasted between 30 and 60 min and were digitally recorded. They were conducted either in-person or via phone calls and complemented by interviewer field notes until data saturation: when a repetition of themes emerged and limited new information was being gathered through additional interviewing [35].

### Data management and analysis

Concurrent with data collection, interviews were transcribed by a professional transcription service. Digital files were reviewed in cases that transcript content was unclear. The study team participated in weekly meetings where early findings were discussed as a group and methodological decisions, including interview guide modification and sampling strategy, were made throughout the study period based on emergent themes. Transcripts were not taken back to the participants, but instead emergent themes were incorporated into new interviews throughout data collection for clarifications and confirmations as member checking [30].

After interviews were conducted and data saturation was reached, transcripts were uploaded to the qualitative data analysis software Dedoose (SocioCultural Research Consultants, Manhattan Beach, CA, USA) for data management and analysis. Two codebooks containing 40 and 24 descriptive codes, were developed for tribal member and leader transcripts, respectively, based on interview guide content and study objectives [36].

Drawing from the analytic approach of Grounded Theory [37, 38] and in a team-based approach, four analysts first conducted initial line-by-line coding and memo-taking to identify tentative categories of inquiry. Codebooks were then modified to better fit the emergent themes and sub-themes. Analytic categories that were most relevant to the research questions were then identified and used as focused codes to separate large chunks of data for interpretation.

**Fig. 1:**
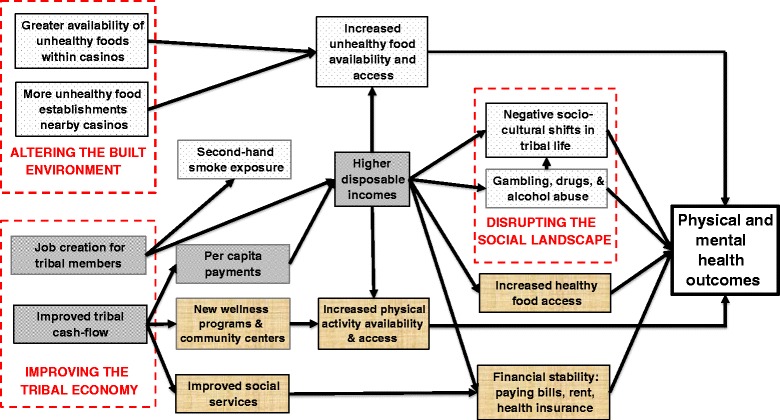
Perceived pathways through which casinos influence health

Second, we used the advanced memos, in combination with the primary themes and sub-themes identified from the previous step, to develop an overarching conceptual framework that represents the findings as a series of perceived determinants of health, organized into three primary pathways (Fig. 1). Finally, using Dedoose, we extracted exemplar quotes to illustrate key findings [39].

## Results

Tribal members described the influence of casinos as a microcosm of the larger, more dynamic societal systems in which they exist. Perceptions of casinos varied by individual, depending on his or her circumstance. Overall, however, shared perceptions emerged as themes and sub-themes from the data and could best be organized into and interpreted as three overarching pathways by which casinos may impact health outcomes through positive and negative health determinants (Fig. 1). The three main pathways through which casinos were perceived to influence health included the following: 1) Improving the Tribal Economy, 2) Altering the Built Environment, and 3) Disrupting the Social Landscape. Below we describe in detail the findings related to each pathway and the key factors along these pathways that may influence health (labeled “health determinants” and italized in the text).

### Pathway 1: Pathways to health through improving the tribal economy

Tribal members described casinos to most directly influence health through a combination of both positive and negative health determinants stemming from changes to the tribal economy. *Job creation for tribal members* and *improved tribal cash flow* were the primary distal health determinants forming this economic pathway. *Per capita payments*, *higher disposable incomes*, *new physical activity programs & community centers, and improved social services* also emerged as health determinants along this pathway.

#### Job creation for tribal members

Tribal members explained job creation to be an important contribution of casinos and a positive influence on individual emotional health. However, the level of enthusiasm toward job creation depended on the size of the casino that was within any one respondent’s community. The tribal member below explained the large number of jobs created in her community:

*“So, that (getting a job) was a big plus…There’s also a golf course and it also has barns…that opened employment too. So a lot of (jobs), not just from the casinos. The casino alone has 600 employees.”*

-Tribal member, large casino, southern California

By contrast, the tribal member’s quote below represents someone from a much smaller casino with a more tempered opinion on the topic.

*“Well, I guess some more people are working…There’s not a whole lot of tribal members working there like you’d think.”*

-Tribal member, small casino, northern California

Despite varying levels of enthusiasm toward job creation by casino size and individual participant, overall it was perceived to be an important contributor to better community health due primarily to the higher disposable incomes made available to tribal members who found casino employment. Money earned from working in the casino was described by participants as a much-needed contribution to their household and individual financial stability, using paychecks from casino work to pay life expenses, including bills and rent.

However, several tribal members highlighted the occupational hazard posed by working in a casino due to *second-hand smoke exposure*. Members discussed their disgust for cigarette smoke in casino environments and their concern for the occupational health of employees.

*“Yeah, they do have employment opportunities, only that I don’t like working around smoke…So I refuse to work there, because I love my lungs.”*

-Tribal member, large casino, southern California

Both tribal members and leaders described the economic stimulus of the casino to impact community health distally through *job creation*, and proximally through additional income earned from working those jobs, allowing for increased *financial stability: paying bills, rent, health insurance*. At the same time, the occupational hazard of *secondhand smoke exposure* was raised as a negative aspect related to empolyment at a casino.

#### Improved tribal cash-flow

Casino-generated profits, which vary substantially in size by tribe, typically flow directly to tribal councils (after lenders and any co-owners have been paid) who make decisions about their distribution. In our sample, participants explained that both profits from casinos, as well as distributions from the RSTF (for smaller casinos and non-gaming tribes), are used for *per capita payments*, *improved social services*, and building *new wellness programs and community centers*. Despite differences in profit size by casino size, the majority of interviewees perceived *improved tribal cash flow* positively. However, salient negative viewpoints also emerged with regard to casino-related *per capita payments*.

#### *Per capita payments* result in *Higher disposable incomes*

*Per capita payments*, stemming from casino profits (either directly or through the RSTF) were described as dividend payments that many tribal councils elect to distribute amongst community members in order to comply with the federal mandate that casino profits be reinvested in tribal welfare or as a way to satisfy tribal members’ requests to receive economic benefits from gaming on either their lands or from the RSTF. During interviews, *per capita payments* were discussed at length as mechanisms of wealth redistribution that were perceived to contribute to the positive physical and emotional health of individuals by contributing to *higher disposable incomes*.

Positive perceptions largely stemmed from individuals who were currently receiving *per capita payments* of substantial value. Tribal leaders and members explained that *per capita payments* have allowed many people “*to get caught up with bills*” and more easily afford to rent/live in their houses. “*They gave us a roof over our head,*” said one tribal member. Further, participants described using the dividend money to now buy foods perceived to be healthier for their families.

“*I don’t know how that affects body mass index, but I know I’ve been very fortunate to buy my children as much organic food as I can with help from per capita.*”

-Tribal leader, small casino, northern California

Tribal members, especially those in larger casinos with more economic resources, also described *per capita payments* as contributors to *increased physical activity availability and access*.

*“A lot of our kids will participate in football season…they’ll actually participate on those (types of) teams where maybe before they weren’t able to because their parents couldn’t afford to pay…”*

-Tribal member, large casino, southern California

Negative perceptions of *per capita payments* primarily were voiced by tribal members who had experienced payment decreases over time or were no longer receiving payments at all. Those tribal members who discussed casino profits in a negative light did so because they felt they were not receiving enough of their fair share, or were unclear how their particular tribal council made decisions related to utilization of that money, suggesting a lack of leadership transparency.

Tribal leaders discussed *per capita payments* very cautiously, explaining that there are prominent examples of money misuse within communities, ascribing blame to payments for facilitating *gambling, drugs, and alcohol abuse*.

*“Yeah, we have a lot of drug addicts that are hanging out more. Some of the houses that have always kind of been druggie homes, but more now because that casino is there…we see a lot of that.”*

-Tribal leader, small casino, central California

Moreover, not all participants agreed that *higher disposable incomes* were only being used positively for food purchasing. One leader acknowledged the *increased healthy food access* thanks to higher incomes, but lamented, “*They do (have better access to healthier food), but they don’t use it, I guess.*” Overall, interview data revealed mixed opinions toward the impacts of *per capita payments*.

#### *New wellness programs & community centers* support *increased physical activity availability & access*

While tribal members were generally unsure to what extent improvements in community services could be attributed specifically to the use of *improved tribal cash flow*, most participants, especially those in larger tribes, did describe *new wellness programs & community centers* since the casinos were built. Most notably, tribal members explained increased opportunities for physical activity through the implementation of sports and fitness programs, as well as subsidies provided for their children’s participation in those types of programs.

*“It (casino) provides the sports, and then there’s the transportation. There’s the equipment. There’s the coaching. All that stuff that goes along…the recreation…so it provides all of that.”*

-Tribal member, large casino, southern California

Moreover, interviewees spoke positively about the use of *improved tribal cash flow* as a funding stream for making structural changes to improve health, highlighting the construction of new community centers and sports facilities, such as gymnasiums and wellness centers, offering sports and fitness classes to adults and children alike.

*“We were also able to build a fitness center…we have exercise equipment, we have a pool…it can turn into volleyball there. We’ve got exercise classes, like Zumba. So it has gone a lot of good things for people.”*

-Tribal member, large casino, southern California

These infrastructure additions, coupled with the related wellness and sports programs, were perceived to contribute positively to children’s physical health and was a more salient perception among participants of larger-sized casinos with bigger gaming profits.

#### *Improved social services* contribute to *financial stability*

There was also acknowledgment by participants that *improved tribal cash flow* was used for *improved social services,* subsidizing or paying for the health insurance of tribal members, as well as creating scholarship funds of college-seeking tribal members. In addition, some tribal communities use profits to provide meal tickets for elderly tribal members. In other cases, profits are allocated as cash on a case-by-case basis for individuals or families facing tough times. Not all tribal communities reported receiving such a large range of social service benefits from *improved tribal cash flow* though, and perceptions varied by individual. Because tribal councils set priorities and have autonomy to determine how to use gaming or RSTF monies based on their own unique community characteristics, tribal member perceptions also varied by community.

### Pathway 2: Pathways to health from altering the built environment

Casinos, themselves, are establishments that offer a new social environment for tribal and non-tribal members to experience. In relation to health, interview data revealed structural changes to the surrounding built environment as a result of gaming operations. Positive changes to the physical activity environment, funded by *improved tribal cash-flow*, included the construction of new community centers/facilities and was described above. However, gaming operations were perceived to negatively change the food environment, by offering a *greater availability of unhealthy foods within casinos*, as well as spawning *more unhealthy food establishments nearby casinos*. However, interviewees expressed mixed opinions toward the influence of these environmental changes on actual health-related behaviors.

#### Greater availability of unhealthy foods within casinos

Participants first discussed the increase in unhealthy food options available to them as a result of casinos in comparison to those of the past, pointing out that today, “*there’s really not a lot of healthy choices*” being offered, including mostly “*fried food and deep fried food*”. One tribal member added, “*Food-wise, casinos have great buffets!*” Despite such reports, participants were reluctant to solely place blame on casinos for negatively influencing health. They emphasized the responsibility of parents, not casinos, for influencing household and individual dietary behaviors.

*“I think it’s what you prepare your kids, you know? Maybe the person doesn’t know how to cook and they’re feeding hamburgers or Hamburger Helper…so I think it’s more parents influencing kids versus the casino when it comes to food…”*

-Tribal member, large casino, southern California

Tribal members put the onus on themselves, as parents and household units, to ensure that their children eat “healthy” foods. In fact, as described above, some tribal members suggested to the contrary—that the *per capita payments* actually allowed them to make healthier food choices while shopping.

*“Oh, my health, yeah it (the casino) does play a role in health because I’m able to provide…I’m able to take my kids places and show them things, pay for outings and what not. I mean, we’re able to buy better food.”*

-Tribal member, large casino, southern California

In terms of the perceived physical health impact as a result of food environment changes, community member views toward casinos were not negative, especially for children.

#### More unhealthy food establishments nearby casinos

Tribal leaders and members noted the influx of new food establishments as a result of casino development. These new establishments took a variety of forms, including new restaurants, as part of, or nearby the casinos, as well as new fast-food chains and carry-out restaurants. Because of *higher disposable income*s from *job creation for tribal members* or *per capita payments*, individuals explained how some households are able to eat out more often than they would have previously. Comparatively, though, foods at casinos are expensive so surrounding food establishments are more often accessed than those in casinos.

“…*They’d* (tribal members) *rather go to McDonald’s where it’s cheaper, or somewhere it’s cheap compared to the casino, where it’s expensive. So it’s* (casino development) *contributing to that* (eating out at fast-food).”

-Tribal member, medium-sized casino, central California

Overall, interviews suggest that *increased unhealthy food availability and access* is associated with gaming operations. Participants had mixed opinions toward their actual impact on food-related behaviors and health, though.

### Pathway 3: Pathways to health by disrupting the social landscape

The perceived impact of casinos on the social fabric of communities was a strong, underlying theme that is difficult to disentangle from the factors through which a stronger tribal economy and casino environment are perceived to impact health. Interview data indicate that some tribal leaders, more so than do tribal members, point to the unhealthy social environments of casinos as facilitators of *gambling, drugs, and alcohol abuse*.

Casinos were perceived to change the social landscape by introducing a combination of money, “*outsiders*”, gambling, and drug and alcohol abuse. Data suggest that casino money creates a “*culture of dependency*” as a result of *per capita payments* in some cases, and a “*loss of tribal unity*” in others. With reference to casino money and greed, one tribal leader explained that there is a disconnect between casino life and traditional tribal culture.

*“That’s what the casino did to our people…it just made them move away from the spiritual walk and it made them start thinking more about the wrong things to really think about.”*

-Tribal member, small casino, central California

Participants did not articulate direct impacts of casinos on individual health in relation to these larger societal shifts, but did allude to the negative atmosphere of the larger casino culture while discussing such issues.

*“Well, yeah because you know there are drug users and… the environment of being able to smoke and drink. And hang out… what people do on drugs. They don't really have a responsibility…just sit around and some may do stuff with it and some may not…an environment that allows you to come in there, you can smoke, you can stay. You don't really have to have any business other than coming and people find that as a past time…people on drugs…can use the Casino as a way just to get away, almost just like drugs. So, just to get, you know, do something and be in the zone.”*

*-Tribal leader, small casino, central California*

Underlying these prominent perceptions were descriptions by interviewees about social concerns of disenfranchisement, lack of tribal sovereignty, and feelings of entitlement by “*the younger generation*”. While this was not the majority view expressed, such feelings were strong enough among a small sample of community members and tribal leaders who described their retracted support of casino development while speaking with hindsight.

*“If I could travel back in time, I would try to stop the casino from being built because I would rather be more happy being broke than having a little bit of extra money”.*

*-Tribal member, large casino, central California*

Overall, a small subset of participants described strong feelings that the casino negatively contributed to *disruptions in the social landscape* of tribal communities.

## Discussion

By conducting semi-structured interviews among American Indian tribal members and leaders throughout California, we were able to generate an understanding of how this population perceived gaming operations to impact their environment, behaviors, and health. Participants in this study perceived casinos to both positively and negatively impact several important determinants of their community and individual health, through economic, environmental, and social pathways.

### Pathways to health through improving the tribal economy

Most notably, tribal leaders and members described the gaming operations and their stimulation to the tribal economy positively, highlighting *increased job creation* and *improved tribal cash-flow*, which tribal councils reportedly allocate toward *per capita payments*, *new wellness programs & community centers*, and for *improved social services*, such as health insurance subsidies.

Tribal members underscored the positive impact of *per capita payments* on individual and household levels of *disposable income*, which participants said have allowed them and their children to more easily engage in positive-health behaviors. This finding is aligned with quantitative estimates of improved health indicators and health-related behaviors corresponding with improved tribal member income from American Indian gaming operations between 1988 and 2003 nationwide [27]. Improvements in physical and emotional health, in particular, and as a result of increased economic resources, are plausible considering that tribal members in our study discussed their use of casino profits for paying bills, acquiring better or more stable housing, buying healthier foods, such as “*organic products*”, and enrolling their children in sports. The positive health behaviors reported among our study sample are consistent with findings that a casino presence on tribal lands was associated with decreased probability of overweight among American Indian children, also in California, and with findings of reduced probability of obesity and diabetes among American Indian adults nationwide [9, 21].

On the other hand, tribal members and leaders stressed that *higher disposable incomes* from *per capita payments*, as opposed to *higher disposable incomes* from *job creation*, may also negatively influence individual health behaviors. For example, excess drinking and drug abuse were frequently noted as unintended behavioral consequences from *per capita* payments with direct negative consequences for the physical and mental health of communities. These findings are consistent with ethnographic research conducted by Bruckner and colleagues [11] who noted that participants from the Eastern Band of Cherokee Indian community frequently cited spending per capita payments on drugs and alcohol, as well as on new motor vehicles. The authors speculate that the combination of these activities could be producing more accidental deaths in association with the timing of casino payments. However, other research from a nationwide sample showed that smoking and heavy alcohol use actually decreased in association with higher incomes from gaming [9].

Tribal leaders in our study perceived the casino environment to be largely unhealthy, and as a facilitator of *gambling, drugs, and alcohol abuse*. These observations are supported by data showing that neighborhood access to gambling opportunities was associated with an increased odds of gambling behaviors, over and above individual characteristics, although these findings where not specific to American Indians [40]. Gambling is a known risk factor for a variety of mental and physical health conditions [41]. And although *job creation for tribal members* was perceived positively by our study participants, a salient concern emerged in relation to worries about *second-hand smoke exposure* as a result of finding a casino job. Secondhand smoke is a major occupational hazard in casino environments where increased levels of it exist, and where elevated levels of tobacco-specific biomarkers in non-smokers’ blood, urine, and saliva have been found [42, 43].

To explain these discrepant findings, it is important to consider that the emotionality of one’s experience is a key factor influencing memory and recall [44]. So because the purpose of our qualitative interviews was to gather in-depth personal experiences and narratives [30, 32], participants potentially were more apt to discuss their own memorable experiences during the interviews. While such findings are reflective of individual-level perceptions, and can be useful for informing policy planning, they may not be reflective of overall population trends and likely vary by individual and tribal characteristics. Also, individual perceptions of casino benefits and costs is not a straightforward issue: Casino development data suggest that tribal members who are likely to receive substantial personal benefits from gambling development, such as new construction of a wellness center or hefty per capita payouts, are more likely to believe that positive benefits outweigh costs [45]. This fact is pertinent to our study because some respondents received larger positive economic-related benefits than others due to their specific community and individual situations, thus likely influencing their perceptions toward gaming and narratives during our interviews. However, even more disparate findings may be found in other states where redistribution policies (e.g., RSTF) are not in place to provide non-gaming tribes with similar benefits from gaming. At the same time, seeing one friend or family member turn to drug use as a result of increased disposable income can stand out as a salient experience for an entire community despite the vast majority of other community members not having the same experience.

### Pathways to health from altering the built environment

A number of changes to the built environment, most prominently characterized by improvements to the physical activity environment were described. Specifically, participants perceived improvements to programs and services, as well as to community centers and health and wellness facilities as a result of casino development. If true, then this finding has positive implications for community health and makes a strong argument for gaming operations to play a positive role in addressing chronic disease prevention efforts, such as those for diabetes, on tribal lands. Barriers to physical activity among American Indian children have been reported in qualitative studies to include a lack of access to and availability of health and wellness facilities, adequate equipment, and trained staff personnel [46, 47]. Tribal members in our study highlighted the use of *improved tribal cash flow* for improving the physical activity environment by addressing all of these barriers, at least among participants of casinos with greater economic resources (i.e., >1,000 slots).

This finding has positive implications for child health on tribal lands. Gordon-Larsen and colleagues [48] illustrated an association between community availability of physical-activity facilities in the built environment and disparities in population activity and overweight patterns. Among American Indian school-aged children, higher level physical activity levels in second grade have been shown to be associated with lower levels of percentage body fat among the same children in fifth grade [49], an example from the larger body of well-established literature illustrating important physical health benefits from physical activity during childhood.

On the contrary, tribal members in our study perceived changes to their food environments from casinos that offer *more unhealthy food establishments nearby casinos* and *greater availability of unhealthy foods within casinos*. Bachar and colleagues [50] found similar changes to the food environment among the Eastern Band of Cherokee Indians in North Carolina. They concluded that as family income increased from casinos, the array of fast food choices also increased, with 19 fast food restaurants available in just 3 miles of one district center. So our findings, and those of Bachar et al. [50], agree that although casinos and casino profits can have positive impacts on individual or household incomes, they may also have negative effects on the food environment and food-related behaviors due to additional disposable income available for eating out.

However, it is noteworthy that in our data set, participants indicated that while disposable incomes rose as a result of *per capita payments*, they were not only used for eating out but also for making healthier food-related decisions, such as “*buying organic*.” In fact, in our study, parents largely put the onus on good parenting to ensure their children’s healthful dietary behaviors, a finding similar to one by Akee and colleagues [51], who believed parental quality to be a mechanism for better educational attainment and decreased chances of committing a crime in the face of casino environments. Good parenting has been shown to be a protective factor from several health issues important to good adolescence development [52], including weight and weight-related behaviors [53] as well as tobacco and alcohol abuse [54]. Also, because the local food environment of a community is associated with indicators of nutritional status, such as obesity prevalence [55], there is a need for additional research in other settings to understand how casinos may be modifying those food environments, related dietary behaviors, and nutritional health of tribal members in tribes with similar characteristics.

### Pathways to health by disrupting the social landscape

Participant explanations of increased drug and alcohol abuse, troublesome gambling behaviors, and unhealthy casino environments were focused on individual-level health impacts. The emergent themes in this pathway may be due in part to the memorable and emotion-evoking impressions left by vivid accounts of alcohol and drug abuse on individual memories. There is evidence to suggest that one’s perceived likelihood of risk is related to personal experience [56], which people typically use as a heuristic to filter information and evaluate risks, health or otherwise [57], and likely emerged so explicitly during interviews for that reason.

However, casino impacts on social health were also expressed strongly by some participants who provided insights about their communities as a whole. Our data are in line with other research that examined community leader perceptions of social and economic impacts on tribal communities, finding that overall economic impacts were perceived positively, but social concerns were important to people [58]. For instance, in our study, tribal leaders suggested that large *per capita payments* contributed to attitudes of entitlement and general dependency on handouts, negatively impacting the younger generation. Negative perceptions do not always exist in relation to casino development though. Momper and Dennis [59] found that community-level relations between tribal members and non-members improved as a result of casino operations opening on tribal land in the United States Midwest. Our data did not reveal any emergent themes in relation to such relationships; however, our semi-structured interview guide did not have a focus on this particular topic either.

Some tribal members also talked about feelings of cultural identity loss, in part, due to gaming activities, comparing the current casino environment to the past when people would help each other more often in a more unified community dynamic. This finding is similar to that from a case study examining the social impact that American Indian gaming had on one reservation in Minnesota, where tribal members expressed concern that American Indian values were being replaced by materialism [60]. Because data suggest that the traditional cultural identify of American Indians may be a protective factor for gambling behaviors and alcohol abuse [61], our findings about identity loss may highlight shifting social norms and casino environments where negative health-related behaviors are more prevalent. This phenomenon will have to be studied in more detail, however, to more fully understand the relationship between cultural identity and practices in relation to casino development and tribal community health. Understanding unique cultural constructs of health and the social environment in American Indian communities is important to inform decision making for community-level health prevention [62].

### Limitations

This study has some limitations. First, we employed a stratified purposive sampling strategy in order to identify participants from different geographic regions of California, varying tribal sizes, and gaming or non-gaming affiliation. However, in doing so, the number of participants within some strata is small, despite having a relatively large total sample of participants for this type of qualitative work. For instance, tribal leaders from casinos with fewer economic resources (<350 slots) are better represented and therefore findings may be unique to those types of casinos in this particular state. While this qualitative study did not aim to be generalizable to all tribal communities in California or elsewhere, and should not be interpreted as such, the transferability of our findings may still be further limited to casinos with similar economic resources (and thus community needs) and related gaming operations [63]. Our sample included individuals who lived outside of tribal lands and therefore may have received fewer benefits of the casino; however, we feel this perspective is important to include when considering overall perceptions toward casinos. Finally, the American Indian gaming situation in California is unique in its establishment of the RSTF, whereby even non-gaming tribes are receiving monies from casino profits. This further limits the transferability of our findings, but still can provide useful insights about such programs. Also, findings may have been interpreted differently had we applied methodological triangulation to this study by using another data collection method, such as secondary food environment data, in order to corroborate results and offer potentially new perspectives [31]. It is also possible that participant narratives were somehow shaped by the interviewer affiliation to a school of public health within a university.

## Conclusion

Participant perceptions toward casinos varied by community and individual experience, but overall, salient themes can be organized into and interpreted as three plausible pathways by which casinos may impact health (Fig. 1). Casino development is perceived to have varying effects on tribal community health and wellness, creating economic stimulus, but at the same time causing social disruption and changes to the built environment that impact upon physical activity opportunities and food security. Considering a holistic view of health that includes a dynamic balance of physical, emotional, social, spiritual, and intellectual dimensions [64, 65], gaming operations on tribal lands have wide-reaching impacts that offer both challenges and opportunities for public health improvement. With a better understanding of unique community perceptions toward casino development and its impact on differential dimensions of health, public health policy makers in California, including both federal-level decision makers and tribal council members, can be better informed while designing social policies and setting priorities for resource allocation on tribal lands with gaming operations. Using the proposed model as a starting point for community-level policy development and health prevention planning may be useful for addressing health disparities, by providing a framework to alter the determinants of health impacting American Indian communities, through the voices of the tribal members and leaders themselves.

## Abbreviations

IRB, Institutional Review Board; RSTF, Revenue Sharing Trust Fund
